# Improvement of Motor Imagination and Manual Ability Through Virtual Reality and Selective and Nonselective Functional Electrical Stimulation: Protocol for a Randomized Controlled Trial

**DOI:** 10.2196/63329

**Published:** 2024-11-22

**Authors:** Montserrat Santamaría-Vázquez, J Hilario Ortiz-Huerta, Aitor Martín-Odriozola, Olalla Saiz-Vazquez

**Affiliations:** 1 Health Sciences Department Universidad de Burgos Burgos Spain; 2 Fesia Clinic Donostia-San Sebastián Spain; 3 Fesia Technology S.L. Donostia-San Sebastián Spain; 4 Department of Physiology University of the Basque Country (UPV/EHU) Leioa Spain

**Keywords:** electric stimulation therapy, motor imagery, virtual reality exposure therapy, hand strength, hand injuries

## Abstract

**Background:**

Motor imagery (MI) is a cognitive process that has been shown to be useful in the rehabilitation process after brain injury. Moreover, functional electrical stimulation (FES) and virtual reality (VR) have also been shown to be effective interventions in many parameters, and there is some evidence of their contribution to the improvement of MI capacity.

**Objective:**

This study aimed to compare the improvements in MI parameters, grip strength, and manual dexterity obtained using VR, FES, and selective FES based on multifield electrodes in healthy people.

**Methods:**

This clinical randomized controlled trial (RCT)with 4 branches will involve 80 healthy university students, with blinded third-party assessment. Participants will be divided into 4 groups: control (no intervention), selective FES (Fesia Grasp), traditional FES (Globus Elite), and Virtual Rehab Hands (Leap Motion sensor). Each group will receive 5 daily sessions, and assessments will be conducted at baseline, postintervention, and follow-up. The Movement Imagery Questionnaire-Revised (MIQ-RS) and chronometry will be used to assess MI, strength will be measured with a digital dynamometer, and manual dexterity will be evaluated with the Nine Hole Peg Test (NHPT) and the Box and Block Test (BBT). Statistical analyses will include 2-way repeated-measures ANOVA with post hoc Bonferroni correction to compare group differences over time, with nonparametric tests (eg, Kruskal-Wallis) being used if normality or variance assumptions are violated. The study will be organized into 3 phases: preparation, data collection, and analysis. The preparation phase will involve finalizing project protocols and obtaining ethical approvals. The data collection phase will consist of recruiting participants, randomizing them into 4 intervention groups, and conducting baseline assessments, followed by intervention sessions. Finally, the analysis phase will focus on evaluating the data collected from all groups and compiling the results for presentation.

**Results:**

The study received approval in July 2023, with recruitment and data collection starting in September 2023. The recruitment phase was expected to conclude by July 2024, and the entire study, including the 2-week follow-up, was set to finish in September 2024. As of July 2024, we had enrolled 100% of the sample (N=80 students). We plan to publish the study findings by the end of 2024.

**Conclusions:**

Improvements in MI and upper limb functionality are expected, particularly in the selective FES group. This RCT will identify which intervention is most effective in enhancing these skills, with potential benefits for patients with neurological motor disorders.

**Trial Registration:**

ClinicalTrials.gov NCT06109025; https://clinicaltrials.gov/study/NCT06109025

**International Registered Report Identifier (IRRID):**

DERR1-10.2196/63329

## Introduction

Motor imagery (MI) is defined as the cognitive process of imagining the movement of one’s own body part without actually moving it [1]. The procedure of testing MI via electroencephalography mainly consists of 5 phases: signal data acquisition, data preprocessing, feature extraction, feature classification, and device control interface [2]. The data acquisition phase includes MI signal collection, signal digitization, and data storage. The data preprocessing phase involves filtering, cleaning, and transforming the data. The feature extraction phase extracts discriminative features containing useful information from electroencephalogram signal data. The feature classification phase uses the extracted features as input to train machine learning models. Finally, in the device control interface phase, the categorized signals are translated into commands to control devices, such as robots, home appliances, and wheelchairs [3].

MI can help people with disabilities and older adults to perform specific tasks through imagination without physically performing any limb movement [1]. Considering this, different techniques have been proposed in recent years that could improve MI in individuals with neurological pathologies. Among the most used techniques are mirror therapy, virtual reality (VR), and augmented reality [4]. Although studied less, electrical stimulation has also been shown to help improve MI parameters in such type of pathologies [5].

Neuromuscular stimulation is an application of electrical stimulation used in movement rehabilitation [6]. Functional electrical stimulation (FES) is a subtype of neuromuscular stimulation in which the stimulation assists functional and purposeful movements [7]. Electrical currents are applied to motor nerves, producing muscle contractions in a sequence that allows the patient to perform different tasks. Examples might include grasping a key, holding a toothbrush, standing up, cycling, or walking [8].

FES was developed in the 1960s, emphasizing its potential as an assistive technology [9]. Since then, FES has evolved into an important therapeutic intervention that clinicians can use to help people with neurological diseases to regain motor abilities [10].

FES device technology has traditionally relied on conventional electrodes. However, multifield surface electrodes have emerged, consisting of groups of several small conducting fields, which can be activated/deactivated and configured independently [11].

The Fesia Grasp (Fesia Technology) [12] is the only commercial FES hand rehabilitation device based on multifield electrodes (32 cathodes and 8 anodes). It delivers trains of biphasic pulses to different electrode fields in an asynchronous manner to generate contractions of the forearm muscles (it can trigger at least 8 different flexion and extension movements of the wrist and fingers) in order to restore motor function in persons with neurological injuries [11].

Hand rehabilitation is sometimes a lengthy process, and motivation is crucial to the patient’s outcome. Moreover, the outcome of the treatment depends not only on the rehabilitation process of the practitioner but also on the patient’s motivation to engage in the training [13]. One of the ways to make rehabilitation more attractive is to incorporate game-based protocols, such as 2D games, 3D games, VR games [14], and augmented reality games [15].

Notably, the use of VR in game-based training protocols meets both the physiological and the psychological needs of patients [13]. VR creates a dynamic and motivating environment by merging touch, hearing, and vision, which makes patients more engaged in clinical or home training [14]. This technology could also be useful when improving MI.

The objective of this study was to compare the improvements in MI parameters, strength, and manual dexterity obtained using VR, traditional FES, and selective FES based on multifield electrodes in people without neurological pathologies.

## Methods

### Study Design

This will be a parallel-group, clinical randomized controlled trial (RCT). It will be a single-center study, with blinded third-party assessment (the researchers conducting the evaluations will not be involved in the recruitment or randomization of the sample or in the implementation of the interventions). As the study sample, 4 parallel groups, each comprising 20 university students, are planned (N=80). The Spanish version of OxMaR software (Oxford Medical Simulation) will be used to randomize the study sample. The study design followed SPIRIT (Standard Protocol Items: Recommendations for Interventional Trials) 2013 recommendations (Multimedia Appendix 1) and CONSORT (Consolidated Standards of Reporting Trails) 2010 guidelines (Multimedia Appendix 2) for clinical trial protocols.

The first group will receive an intervention based on multifield FES with the Fesia Grasp device (selective FES multifield), the second group will receive traditional FES using the Globus Elite electrostimulation device (based on conventional electrodes), the third group will receive an intervention based on VR, and, finally, the control group will not receive any treatment.

### Study Population

As this will be a feasibility study, with a short intervention (5 sessions in the same week), the selected study population is healthy students. Paravlic et al [16] analyzed numerous studies measuring the strength and MI in healthy populations, both variables in this work, and we have chosen to replicate the same population of previous works.

Student recruitment will be conducted in 2 ways: First, the study will be presented to all students of the Faculty of Health Sciences via email, outlining the objectives and the most relevant information. In the second stage, the snowball method will be used, allowing recruited students to invite other peers or students from other faculties. Sample size calculation was performed using power analysis with SPSS version 28 software. With a significance level of α=.05, a statistical power of 0.80, and an expected effect size of *P*<.05 based on previous studies, it was determined that at least 18 participants per group would be necessary to detect statistically significant differences. This calculation ensures that the study will have sufficient power to avoid type II errors.

Participants will be included according to these inclusion criteria: (1) be of legal age and provide signed informed consent; (2) not have any pathology in the upper limb, such as tendinitis, edema, or fracture; and (3) have intact skin (without breaks, scratches, cuts, and other types of superficial or deep lesions) on the nondominant arm, where the devices will be placed, if applicable. Exclusion criteria include (1) severe medical problems, (2) use of a pacemaker, (3) pregnancy, (4) peripheral neuropathies, or (5) the presence of other neuromuscular pathologies.

Participants may leave the study in the following cases: at their own request, without providing reasons; if any adverse events occur that prevent them from continuing the study; failure to complete the treatment; or failure to complete the sessions within the schedule established for each one. The reason for withdrawal will be recorded in the database.

### Procedure

**Figure 1:**
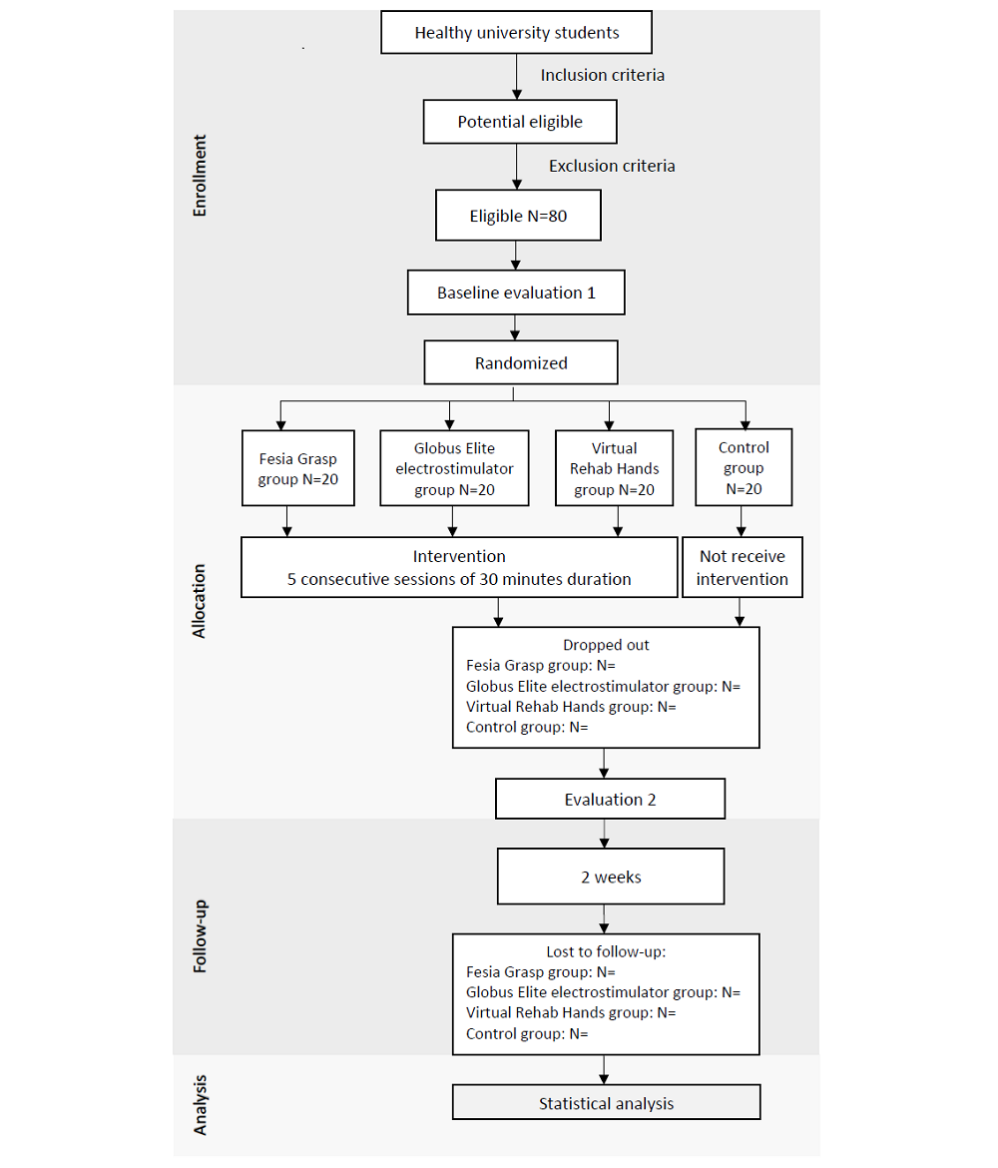
Phases of the project.

There will be 3 phases of the project: preparation, data collection, and a final phase of analysis and reporting (Figure 1). The preparation phase will include drafting the project, obtaining ethics committee approval, and starting sample collection. This phase will last for 2 months. During sample collection, participants will be randomized and assigned to different groups.

The data collection phase will be the longest phase of all and will be spread over 13 weeks. During this period, preintervention assessments, interventions, and postintervention and follow-up assessments 2 weeks after the intervention will be conducted by blind evaluators who will neither know the assigned groups nor participate in the intervention sessions. Each participant will receive the intervention in the nondominant arm.

#### Control Group

This group will not receive any type of intervention, and participants will continue with their usual routine. They will be asked to commit to not start any physical activity other than their usual routine during the study period.

#### Selective FES (Fesia Grasp) Group

For this group, 5 sessions of 30 minutes each will be carried out on consecutive days, and 2 available protocols will be used: habituation and repetitive task training. The habituation protocol will be used during the first half of the first session to allow the participants to adapt to the sensation of electrical stimulation by means of an automatic sweep of electrical stimulation. The repetitive task training will be used in sessions 1-5. First, the device will be configured, selecting cathode combinations that generate the clearest flexion and extension movements of the wrist, thumb, index, and fingers 3-5. If the motor threshold for any of the movements is not found in any of the users, or stimulating any of these movements may be considered harmful or not beneficial, it will not be selected. It will be important to carry out this configuration thoroughly in the first session, and small variations will be made in subsequent sessions. The therapy will then be initiated (to be adjusted according to the progress of the patients).

#### Sessions 1-3

Selective contractions of the forearm muscle groups will be performed. The proposed progression for the sessions is as follows: beginning (5 minutes), which will involve placing the device and checking that the previous configuration is correct. In the first part (5 minutes), selective contractions of the different muscle groups will be performed using FES. In the second part (10-15 minutes), voluntary selective contractions will be performed by the patient, guided by the therapist.

#### Sessions 4-5

These sessions will include training in activities of daily living with the help of FES. The general development proposed is as follows: beginning (5 minutes), which will involve setting up the device and checking that the previous configuration is correct. In the first part (5 minutes), selective contractions of the different muscle groups will be performed using FES. In the second part (10-15 minutes), the previously activated functional movements will be included in functional actions using FES to grasp objects of different sizes.

#### Traditional FES (Globus Elite Electrostimulator) Group

For this group, 5 sessions of 30 minutes each will be carried out on consecutive days. The device will be applied, adjusted according to the progress of the patients.

#### Sessions 1-3

These sessions will consist of general repetitive contractions of the muscle groups of the forearm. The general sequence is, again, as follows: beginning (5 minutes), which will involve setting up the device and checking that the configuration is correct. In the first part (5 minutes), general contractions of the different muscle groups will be performed using FES, while in the second part (10-15 minutes), the patient will perform general voluntary contractions, guided by the therapist.

#### Sessions 4-5

These sessions will also include training in activities of daily living using FES. The general development proposed for the sessions is as follows: beginning (5 minutes), which will involve setting up the device and checking that the configuration is correct. In the first part (5 minutes), general contractions of the different muscle groups will be performed using FES, while in the second part (10-15 minutes), the previously activated functional movements will be included in functional actions using FES to grasp objects of different sizes.

The electroparameters selected will be the same in both FES groups (frequency: 25 Hz; pulse width: 250 µs). Therapy times will vary according to the patient’s fatigue, as measured by the Borg scale. The Borg scale score should not exceed 3 points out of 10 one minute after the end of the exercise. If the perception of fatigue is higher, first the intensity (eg, frequency of movements) and then the duration of therapy will be adjusted.

#### Virtual Rehab Hands Group

The group will play for 5 sessions of 30 minutes each on consecutive days. The Virtual Rehab Hands device includes fine motor games. There is an ergonomic support to rest the arm, and the user is able to perform the exercises more comfortably with the Leap Motion sensor, which accurately detects movements of the hands, fingers, and wrists. Each session will be divided into 3 phases: warm-up, effort, and cooldown. In the warm-up phase, we will play “wrist flexion and extension bird,” “flexion and extension candy,” “ulnar-radial deviation frog,” and “ulnar-radial deviation frog.” In the effort phase, we will play “digital piano,” “balloon tongs,” and “finger abduction.” Finally, in the cooldown (return-to-bed) phase, we will play “frog flexion-extension” and “wrist flexion and extension bird.”

### Measurement Tools

Different assessment tools have been developed that assess hand dexterity, strength, and MI. The Nine Hole Peg Test (NHPT) will be included for the assessment of manual dexterity. The NHPT [17] is a manual test, in which the subject must place 9 pegs into corresponding holes and then remove them, the variable measured being the total time taken to complete the process. Less time implies better manual dexterity.

The Box and Block Test (BBT) consists of a rectangular wooden box with a base 53.7 cm wide and 25.4 cm long, which is divided into 2 square compartments of 25.4 cm on each side, separated by a 15.2-cm-high separator. The test contains 150 cube-shaped wooden blocks of 2.5 cm on each side. The number of blocks the subject has carried from one compartment to another with each hand in 1 minute is recorded. Higher scores indicate better manipulative skills. The hand grip strength (Jamar hand dynamometer) is used to measure grip strength in the hand, allowing the force exerted by the subject to be converted into a numerical reading. The subject is asked to grip the device and perform the maximum sustained contraction. The force is recorded in kilograms. The timing assessment of MI ability requires at least 2 tests. Chronometry measures the temporal congruence between an executed motor act and the same imagined act. In this case, the NHPT itself is proposed as the executed motor act. So, once administered to obtain the test variable (time taken to perform), each participant is asked to imagine the performance of the test, which is timed. Both measurements are taken in seconds to calculate the chronometric ratio (CR) according to the following formula [18]: CR = (time motor act executed – time motor act imagined)/time motor act executed.

Finally, the revised Movement Imagery Questionnaire-Revised (MIQ-RS) [19] consists of 2 subscales, one visual and the other kinesthetic. Each subscale has 7 items, and each item is scored on a 7-point Likert scale (the higher the score, the greater the ease of imagining). For all items, the user is asked to perform a certain motor act (only once), to return to the starting position, and then to imagine it. When scoring the visual scale, the participant is asked to generate an image “as if they could see themselves” doing the gesture, while the kinesthetic scale asks them to “recall the sensation of the movement.” The minimum value is 14, and the maximum value is 98 (minimum 7 and maximum 49 for the subscales). The Spanish version of the MIQ-RS was validated by Cantalejo‐Fernández et al [20] in a sample of university students, achieving Cronbach α values of .90 and a 2-factor structure.

In this study, several devices will be used to carry out the intervention. The Fesia Grasp device bases its operation on superficial electrical stimulation of the antebrachial musculature to generate flexion and extension movements of the wrist, thumb, index finger, and fingers 3-5. The device is Conformité Européenne (CE) marked. The main feature of this device is its multifield electrode, which allows a greater number of movements to be selected, to do so quickly and selectively, and to combine the movements with one another. This is a matrix electrode designed to cover a large portion of the forearm surface in order to stimulate the muscles of the neuromuscular groups of the radial, ulnar, and median nerves. The electrode comprises 32 cathodes (output fields) and 8 anodes (return fields), which can be activated independently or in combination, allowing the electrode to be adapted to the anthropometry of different patients.

Globus Elite 4-channel electrostimulation is a traditional electrical stimulation, which allows up to 8 electrodes to be activated at the same time (4 channels). The device, which is CE marked, allows the use of different types of electrical currents, which are selected according to the desired therapeutic objectives and the evolutionary phase of the patient. Among the currents that can be applied are those also emitted by the Fesia Grasp device. These currents are biphasic and symmetrical rectangular.

Lastly, Virtual Rehab Hands is a therapeutic tool that uses VR for the rehabilitation of a patient with neurological or musculoskeletal disorders. The device provides a wide range of activities designed to improve mobility, coordination, and strength. This tool is based on the idea of gamification in rehabilitation, where patients participate in virtual games that require physical movements to complete challenges. These games are tailored to the individual needs and abilities of each patient, allowing for personalized rehabilitation. Virtual Rehab Hands allows for (1) a comprehensive assessment, (2) personalized treatment planning, and (3) monitoring and tracking of the patient’s progress. This tool has different fine motor games, including 8 video games for fine motor training that can be customized to the needs of each patient.

### Ethical Considerations

This study was approved by the Ethic Committee of Burgos University (IO 2/2023). It will adhere to the Declaration of Helsinki and the requirements established in Spanish legislation. All participants will be provided all relevant information about the study prior to their participation, and signed informed consent will be obtained from them before the evaluation. Data will be anonymized. Once the participants have signed the informed consent form, they will be assigned an alphanumeric code, which only the researcher in charge of anonymizing the data will be able to link. In the database, the alphanumeric code will appear with the rest of the data collected. The linking document between personal data and the assigned code will be stored with a password to limit access. Participants will not receive any compensation for their participation in the study.

This study has been registered with ClinicalTrials (NCT06109025).

### Dissemination Plan

Once the study is completed, we plan to publish the results in high-impact journals, preferably in open access. In addition, we will participate in at least 2 specialized congresses through oral communications or posters.

### Statistical Analysis

IBM SPSS version 28 software will be used for all analyses.

#### General Analysis

Comparative analyses between the 4 groups will be carried out to verify that there are no differences between groups at the beginning of the different interventions (ANOVA for baseline). In addition, descriptive analysis of the sample will be carried out.

#### Inferential Analysis

Two-way repeated-measures ANOVA (group×time) will be used with post hoc Bonferroni correction to test the hypotheses, although the sample does not meet the normality criteria [21]. If the data do not meet the assumptions of homogeneity of variances, the Levene test can also be used to validate this assumption. The use of parametric tests in a nonnormal distribution can be justified in certain cases. In this study, the use of parametric tests has been considered for different reasons, such as their robustness, their greater statistical power, and the homogeneity of variances that exists in the dependent variables of the study. In case the assumptions of normality or equality of variances are not met, nonparametric tests can be used. In this case, the nonparametric equivalent to ANOVA would be the Kruskal-Wallis test, which does not assume normality of data and is appropriate when there are independent groups.

Repeated-measures ANOVA will also be performed, as the use of repeated measures provides a more effective control of extraneous sources of variation associated with individual characteristics (ie, a reduction in error variance is achieved) [22].

## Results

The study received approval in July 2023, with recruitment and data collection starting in September 2023. The recruitment phase was expected to conclude by July 2024, and the entire study, including the 2-week follow-up, was set to finish in September 2024. As of July 2024, we had enrolled 100% of the sample (N=80 students). We plan to publish the study findings by the end of 2024.

As a result of the proposed interventions, an improvement in visual and kinesthetic MI capacity is expected in the groups receiving FES treatment, with a more significant improvement anticipated in the selective FES group. We also expect improvements in the rest of the variables studied: improvement in manual dexterity and manipulative skills and improvement in grip strength. The improvement related to MI is expected to be maintained in the follow-up evaluation. However, regarding the other variables (manual dexterity, manipulative skills, and grip strength), differences are expected between time 1 and time 2 in all of them compared to the control group, with the possibility that these changes may not be maintained over time due to, among other factors, the short intervention period.

## Discussion

### Summary

This study aims to compare the effectiveness of selective FES training, traditional FES training, and VR in improving MI parameters, strength, and manual dexterity in the upper extremities of healthy subjects. For this, 4 groups will be created (selective FES, traditional FES, VR, and control), and the following pre- and posttreatment measurements will be carried out: the NHPT, the BBT, hand grip strength, and the MIQ-RS.

One of the most common sequelae after stroke or other neurological diseases is the limitation or loss of functional capacity of the upper extremities [23]. Around 65% of affected individuals are unable to use their impaired limb in activities of daily living 6 months following the impairment [24], and this loss of functionality directly affects their quality of life, often leading to dependency [25].

MI is a crucial factor in rehabilitating the affected limb, enabling training even when the limb’s motor skills are nearly nonexistent [16]. To carry out interventions based on MI, it is necessary that the person who is going to receive the treatment has a certain facility to imagine these motor acts. This is not always the case, and it is necessary to improve their MI skills so that they can benefit as much as possible from MI-based treatment. Although there are various techniques that have demonstrated good results with regard to improving MI (mirror therapy, for example) [26], more effective and easy-to-use techniques are necessary.

On the one hand, VR has been shown to effectively increase patient motivation and enhance adherence to treatment [27]. If the results in terms of MI improvement are also positive, it could become a technique of choice in people with severely affected limbs in whom following traditional MI treatment is complicated because of a lack of usability.

On the other hand, electrical stimulation is among the therapies with the highest level of evidence for motor rehabilitation of the upper extremities in individuals with neurological conditions [28]. However, its effect on MI parameters is still unknown, as there are few studies in this regard. However, the findings in neuroplasticity parameters suggest that it could be an interesting technique for this [27].

Furthermore, if this FES is applied selectively using multifield electrodes, the effects may be enhanced by concentrating sensory input (both central and peripheral) on the affected muscles and brain or spinal areas, providing the minimal stimulus needed for executing certain motor tasks [29]. In addition to increasing clinical effectiveness, the use of multifield electrodes has been shown to increase the usability of FES systems [30], which could also have an effect on patient motivation and treatment adherence. Should the study demonstrate that FES interventions improve MI, it could present a valuable alternative for enhancing this skill prior to initiating imagination-based treatments, while also improving related skills, such as strength and manual dexterity.

### Limitations

The study has several limitations. First, the subjects may change their physical activity during the week of their participation, and this change may contaminate the data; to avoid this, the contract method has been proposed, so in the informed consent form the participants sign, in addition to their agreement to participate in the study, a commitment not to vary their physical activity is included. However, since the sample comprises healthy subjects, specifically university students, in the event that the results are positive, it is not clear whether they can be extrapolated to healthy older subjects or to persons with neurological pathologies, such as stroke. In both cases, it is more than likely that the number of intervention sessions would have to be increased to find any positive results in the variables studied.

### Conclusion

If the study yields positive results, it would be valuable to test these techniques in individuals with neurological impairments, such as stroke or spinal cord injury, to assess their effectiveness for inclusion in treatment protocols for patients with motor disorders caused by neurological diseases and to support further cost-effectiveness studies.

## Appendix

Multimedia Appendix 1 SPIRIT (Standard Protocol Items: Recommendations for Interventional Trials) 2013 checklist.

Multimedia Appendix 2 CONSORT (Consolidated Standards of Reporting Trails) 2010 checklist.

## Abbreviations

CR: chronometric ratio
BBT: Box and Block Test
FES: functional electrical stimulation
MI: motor imagery
MIQ-RS: Movement Imagery Questionnaire-Revised
NHPT: Nine Hole Peg Test
RCT: randomized controlled trial
VR: virtual reality
